# Ethyl 2-methyl-4-phenyl­quinoline-3-carboxyl­ate

**DOI:** 10.1107/S1600536809018625

**Published:** 2009-05-23

**Authors:** Ayoob Bazgir

**Affiliations:** a Department of Chemistry, Islamic Azad University, Dorood Branch, Dorood 688173551, Iran

## Abstract

In the mol­ecule of the title compound, C_19_H_17_NO_2_, the quinoline ring system is planar [maximum deviation 0.021 (3) Å] and oriented with respect to the phenyl ring at a dihedral angle of 80.44 (4)°. Intra­molecular C—H⋯O inter­actions result in the formation of five- and six-membered rings having planar and envelope conformations, respectively. In the crystal structure, inter­molecular C—H⋯O inter­actions link the mol­ecules into centrosymmetric dimers forming *R*_2_^2^(12) ring motifs. π–π contacts between the rings of the quinoline system [centroid-to-centroid distance = 3.812 (1) Å] may further stabilize the structure. Two weak C—H⋯π inter­actions are also found.

## Related literature

For general background, see: Doube *et al.* (1998 ▶). For ring-motifs, see: Bernstein *et al.* (1995 ▶). For bond-length data, see: Allen *et al.* (1987 ▶).
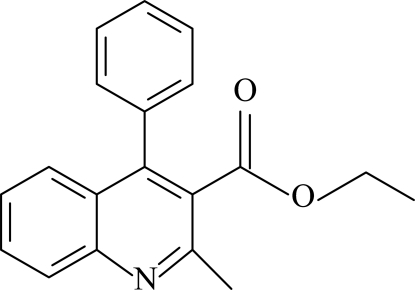


## Experimental

### 

#### Crystal data

C_19_H_17_NO_2_
*M* *_r_* = 291.34
Triclinic, 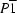

*a* = 9.0282 (10) Å
*b* = 9.362 (1) Å
*c* = 10.7258 (10) Å
α = 69.765 (8)°
β = 66.733 (8)°
γ = 70.605 (8)°
*V* = 761.08 (15) Å^3^
*Z* = 2
Mo *K*α radiation
μ = 0.08 mm^−1^
*T* = 120 K
0.35 × 0.32 × 0.25 mm

#### Data collection

Bruker SMART CCD area-detector diffractometer
Absorption correction: none
8164 measured reflections
3995 independent reflections
3410 reflections with *I* > 2σ(*I*)
*R*_int_ = 0.044

#### Refinement

*R*[*F* ^2^ > 2σ(*F* ^2^)] = 0.065
*wR*(*F* ^2^) = 0.194
*S* = 1.09
3995 reflections
199 parameters
H-atom parameters constrained
Δρ_max_ = 0.33 e Å^−3^
Δρ_min_ = −0.35 e Å^−3^

### 

Data collection: *SMART* (Bruker, 1998 ▶); cell refinement: *SAINT* (Bruker, 1998 ▶); data reduction: *SAINT*; program(s) used to solve structure: *SHELXTL* (Sheldrick, 2008 ▶); program(s) used to refine structure: *SHELXTL*; molecular graphics: *ORTEP-3 for Windows* (Farrugia, 1997 ▶) and *PLATON* (Spek, 2009 ▶); software used to prepare material for publication: *WinGX* (Farrugia, 1999 ▶) and *PLATON*.

## Supplementary Material

Crystal structure: contains datablocks global, I. DOI: 10.1107/S1600536809018625/hk2681sup1.cif

Structure factors: contains datablocks I. DOI: 10.1107/S1600536809018625/hk2681Isup2.hkl

Additional supplementary materials:  crystallographic information; 3D view; checkCIF report

## Figures and Tables

**Table 1:** Hydrogen-bond geometry (Å, °)

*D*—H⋯*A*	*D*—H	H⋯*A*	*D*⋯*A*	*D*—H⋯*A*
C16—H16*A*⋯O1	0.97	2.32	2.711 (3)	103
C19—H19*B*⋯O2^i^	0.96	2.52	3.374 (3)	147
C19—H19*C*⋯O2	0.96	2.59	3.212 (3)	122
C12—H12⋯*Cg*2^ii^	0.93	2.95	3.750 (3)	145
C17—H17*C*⋯*Cg*3^iii^	0.96	2.97	3.883 (3)	160

Symmetry codes: (i) 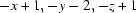; (ii) 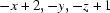; (iii) 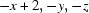. *Cg*2 and *Cg*3 are the centroids of rings C1–C6 and C8–C13, respectively.

**Fig. 1.**
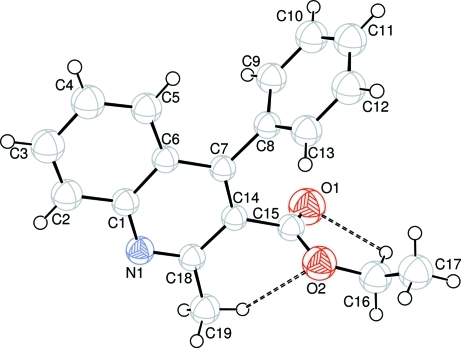
The molecular structure of the title molecule, with the atom-numbering scheme. Hydrogen bonds are shown as dotted lines.

**Fig. 2.**
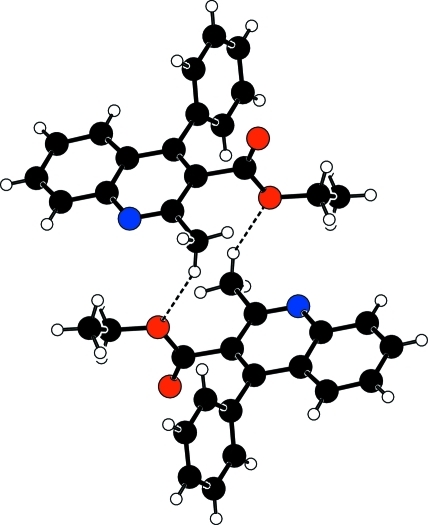
A partial packing diagram of the title compound. Hydrogen bonds are shown as dashed lines.

## supplementary crystallographic information

### Comment

The quinoline moiety is probably the most well known heterocycle, a common and important feature of a variety of natural products and medicinal agents. They have emerged as antimalarial, antiasthmatic, anti-inflamatory, antibacterial, antihypertensive and tyrosine kinase PDGF-RTK inhibiting agents (Doube *et al.*, 1998). Moreover, polyquinolines are found to undergo hierarchical self-assembly into a variety of nano and *meso* structures with enhanced electronic and photonic functions. We report herein the synthesis and crystal structure of the title compound.

In the molecule of the title compound (Fig. 1), the bond lengths (Allen *et al.*,  1987) and angles are within normal ranges. The quinoline ring system A (N1/C1–C7/C14/C18) is planar with a maximum deviaton of -0.021 (3) Å for atom C18, and oriented with respect to the phenyl ring B (C8–C13) at a dihedral angle of A/B = 80.44 (4)°. Intramolecular C—H···O interactions result in the formations of five- and six-membered rings C (O1/O2/C15/C16/H16A) and D (O2/C14/C15/C18/C19/H19C). Ring C is planar, while ring D adopts envelope conformation, with atom O2 displaced by -1.166 (4) Å from the plane of the other ring atoms.

In the crystal structure, intermolecular C—H···O interactions (Table 1) link the molecules into centrosymmetric dimers forming R_2_^2^(12) ring motifs (Fig. 2) (Bernstein *et al.*,  1995), in which they may be effective in the stabilization of the structure. The π–π contact between the rings of the quinoline ring system, Cg1···Cg2^i^ [symmetry code: (i) 1 - x, -1 - y, 1 - z, where Cg1 and Cg2 are centroids of the rings (N1/C1/C6/C7/C14/C18) and (C1–C6), respectively] may further stabilize the structure, with centroid-centroid distance of 3.812 (1) Å. There also exist two weak C—H···π interactions (Table 1).

### Experimental

For the preparation of the title compound, a mixture of ethyl acetoacetate (0.13 g, 1 mmol), (2-aminophenyl)(phenyl)methanone (0.20 g, 1 mmol) and *p*-toluene sulfonic acid (0.1 g, 5.8 mmol) in water (5 ml) was stirred at reflux for 4 h. After completion of reaction (monitored by TLC) the reaction mixture was filtered and the precipitate washed with water (15 ml) and then recrystallized from EtOH/water (1:2) to afford the pure product (yield; 75%, 0.218 g).

### Refinement

H atoms were positioned geometrically, with C—H = 0.93, 0.97 and 0.96 Å for aromatic, methylene and methyl H, respectively, and constrained to ride on their parent atoms, with U_iso_(H) = 1.2U_eq_(C).

### Crystal data

**Table:**

C_19_H_17_NO_2_	*Z* = 2
*M**_r_* = 291.34	*F*(000) = 308
Triclinic, *P*1	*D*_x_ = 1.271 Mg m^−^^3^
Hall symbol: -P 1	Mo *K*α radiation, λ = 0.71073 Å
*a* = 9.0282 (10) Å	Cell parameters from 1548 reflections
*b* = 9.362 (1) Å	θ = 2.4–29.2°
*c* = 10.7258 (10) Å	µ = 0.08 mm^−^^1^
α = 69.765 (8)°	*T* = 120 K
β = 66.733 (8)°	Block, colourless
γ = 70.605 (8)°	0.35 × 0.32 × 0.25 mm
*V* = 761.08 (15) Å^3^	

### Data collection

**Table:**

Bruker SMART CCD area-detector diffractometer	*R*_int_ = 0.044
φ and ω scans	θ_max_ = 29.2°, θ_min_ = 2.4°
8164 measured reflections	*h* = −12→12
3995 independent reflections	*k* = −12→12
3410 reflections with *I* > 2σ(*I*)	*l* = −13→14

### Refinement

**Table:**

Refinement on *F*^2^	0 restraints
Least-squares matrix: full	H-atom parameters constrained
*R*[*F*^2^ > 2σ(*F*^2^)] = 0.065	*w* = 1/[σ^2^(*F*_o_^2^) + (0.0955*P*)^2^ + 0.1587*P*] where *P* = (*F*_o_^2^ + 2*F*_c_^2^)/3
*wR*(*F*^2^) = 0.194	(Δ/σ)_max_ = 0.01
*S* = 1.09	Δρ_max_ = 0.33 e Å^−^^3^
3995 reflections	Δρ_min_ = −0.35 e Å^−^^3^
199 parameters	

### Special details

**Table:**

Geometry. All e.s.d.'s (except the e.s.d. in the dihedral angle between two l.s. planes) are estimated using the full covariance matrix. The cell e.s.d.'s are taken into account individually in the estimation of e.s.d.'s in distances, angles and torsion angles; correlations between e.s.d.'s in cell parameters are only used when they are defined by crystal symmetry. An approximate (isotropic) treatment of cell e.s.d.'s is used for estimating e.s.d.'s involving l.s. planes.

### Fractional atomic coordinates and isotropic or equivalent isotropic displacement parameters (Å^2^)

**Table:**

	*x*	*y*	*z*	*U*_iso_*/*U*_eq_	
O1	0.6903 (3)	−0.70878 (17)	0.11893 (14)	0.0879 (5)	
O2	0.6965 (2)	−0.95151 (14)	0.24871 (13)	0.0696 (4)	
N1	0.44256 (15)	−0.71135 (15)	0.57975 (13)	0.0470 (3)	
C1	0.56342 (18)	−0.68228 (16)	0.60840 (15)	0.0438 (3)	
C2	0.5192 (2)	−0.6418 (2)	0.73600 (17)	0.0551 (4)	
H2	0.4104	−0.6328	0.7953	0.066*	
C3	0.6347 (3)	−0.6159 (2)	0.77284 (19)	0.0622 (5)	
H3	0.6046	−0.5907	0.8575	0.075*	
C4	0.7988 (2)	−0.6273 (2)	0.6834 (2)	0.0615 (4)	
H4	0.8767	−0.6096	0.7094	0.074*	
C5	0.8456 (2)	−0.66413 (19)	0.55808 (18)	0.0523 (4)	
H5	0.9545	−0.6702	0.4994	0.063*	
C6	0.72854 (17)	−0.69295 (15)	0.51769 (15)	0.0420 (3)	
C7	0.76870 (17)	−0.73393 (15)	0.38991 (14)	0.0402 (3)	
C8	0.94211 (17)	−0.75008 (16)	0.29187 (15)	0.0423 (3)	
C9	1.0023 (2)	−0.61966 (19)	0.20110 (18)	0.0546 (4)	
H9	0.9326	−0.5206	0.1985	0.066*	
C10	1.1658 (2)	−0.6361 (2)	0.11427 (19)	0.0619 (4)	
H10	1.2049	−0.5483	0.0535	0.074*	
C11	1.2697 (2)	−0.7819 (2)	0.11829 (18)	0.0612 (5)	
H11	1.3796	−0.7925	0.0614	0.073*	
C12	1.2116 (2)	−0.9125 (2)	0.20633 (19)	0.0622 (4)	
H12	1.2818	−1.0112	0.2081	0.075*	
C13	1.0480 (2)	−0.89658 (18)	0.29248 (17)	0.0526 (4)	
H13	1.009	−0.9851	0.3512	0.063*	
C14	0.64476 (17)	−0.76184 (16)	0.36344 (14)	0.0410 (3)	
C15	0.67936 (18)	−0.80112 (17)	0.22859 (15)	0.0451 (3)	
C16	0.7336 (3)	−1.0130 (2)	0.12943 (19)	0.0640 (5)	
H16A	0.7594	−0.9331	0.0428	0.077*	
H16B	0.6389	−1.0461	0.1365	0.077*	
C17	0.8780 (3)	−1.1482 (3)	0.1307 (2)	0.0694 (5)	
H17A	0.8511	−1.2265	0.2168	0.083*	
H17B	0.9711	−1.114	0.1231	0.083*	
H17C	0.905	−1.1913	0.0532	0.083*	
C18	0.48148 (18)	−0.75086 (17)	0.46289 (15)	0.0441 (3)	
C19	0.3470 (2)	−0.7867 (2)	0.43737 (19)	0.0577 (4)	
H19A	0.2567	−0.6965	0.4356	0.069*	
H19B	0.3086	−0.8727	0.511	0.069*	
H19C	0.3897	−0.8138	0.3491	0.069*	

### Atomic displacement parameters (Å^2^)

**Table:**

	*U*^11^	*U*^22^	*U*^33^	*U*^12^	*U*^13^	*U*^23^
O1	0.1583 (17)	0.0598 (8)	0.0463 (7)	−0.0329 (9)	−0.0344 (9)	−0.0036 (6)
O2	0.1184 (12)	0.0481 (6)	0.0487 (6)	−0.0242 (7)	−0.0284 (7)	−0.0121 (5)
N1	0.0443 (6)	0.0499 (7)	0.0454 (6)	−0.0124 (5)	−0.0135 (5)	−0.0091 (5)
C1	0.0471 (7)	0.0398 (6)	0.0437 (7)	−0.0072 (5)	−0.0161 (6)	−0.0096 (5)
C2	0.0607 (9)	0.0556 (9)	0.0460 (8)	−0.0086 (7)	−0.0143 (7)	−0.0166 (6)
C3	0.0786 (12)	0.0623 (10)	0.0558 (9)	−0.0085 (8)	−0.0288 (9)	−0.0248 (8)
C4	0.0684 (11)	0.0631 (10)	0.0709 (11)	−0.0092 (8)	−0.0375 (9)	−0.0251 (8)
C5	0.0494 (8)	0.0540 (8)	0.0629 (9)	−0.0071 (6)	−0.0261 (7)	−0.0203 (7)
C6	0.0437 (7)	0.0376 (6)	0.0472 (7)	−0.0052 (5)	−0.0191 (6)	−0.0117 (5)
C7	0.0408 (6)	0.0364 (6)	0.0433 (7)	−0.0082 (5)	−0.0146 (5)	−0.0087 (5)
C8	0.0411 (6)	0.0440 (7)	0.0442 (7)	−0.0097 (5)	−0.0151 (5)	−0.0119 (5)
C9	0.0563 (9)	0.0464 (8)	0.0594 (9)	−0.0154 (6)	−0.0170 (7)	−0.0090 (6)
C10	0.0627 (10)	0.0744 (11)	0.0509 (9)	−0.0347 (9)	−0.0119 (7)	−0.0071 (8)
C11	0.0454 (8)	0.0894 (13)	0.0472 (8)	−0.0166 (8)	−0.0103 (6)	−0.0190 (8)
C12	0.0497 (9)	0.0676 (10)	0.0589 (9)	0.0023 (7)	−0.0163 (7)	−0.0192 (8)
C13	0.0499 (8)	0.0455 (7)	0.0544 (8)	−0.0070 (6)	−0.0147 (6)	−0.0086 (6)
C14	0.0442 (7)	0.0394 (6)	0.0405 (6)	−0.0110 (5)	−0.0151 (5)	−0.0075 (5)
C15	0.0476 (7)	0.0471 (7)	0.0435 (7)	−0.0142 (6)	−0.0163 (6)	−0.0089 (5)
C16	0.0854 (13)	0.0621 (10)	0.0558 (9)	−0.0166 (9)	−0.0259 (9)	−0.0238 (8)
C17	0.0650 (11)	0.0801 (13)	0.0642 (11)	−0.0162 (9)	−0.0143 (9)	−0.0266 (9)
C18	0.0433 (7)	0.0452 (7)	0.0439 (7)	−0.0124 (5)	−0.0162 (5)	−0.0060 (5)
C19	0.0497 (8)	0.0728 (11)	0.0576 (9)	−0.0221 (8)	−0.0213 (7)	−0.0115 (8)

### Geometric parameters (Å, °)

**Table:**

C1—N1	1.3708 (19)	C11—H11	0.93
C1—C6	1.415 (2)	C12—C13	1.388 (2)
C1—C2	1.415 (2)	C12—H12	0.93
C2—C3	1.365 (3)	C13—H13	0.93
C2—H2	0.93	C14—C18	1.433 (2)
C3—C4	1.403 (3)	C14—C15	1.5031 (19)
C3—H3	0.93	C15—O1	1.1841 (19)
C4—C5	1.371 (2)	C15—O2	1.3132 (19)
C4—H4	0.93	C16—O2	1.457 (2)
C5—C6	1.417 (2)	C16—C17	1.485 (3)
C5—H5	0.93	C16—H16A	0.97
C6—C7	1.4268 (19)	C16—H16B	0.97
C7—C14	1.3775 (19)	C17—H17A	0.96
C7—C8	1.4939 (19)	C17—H17B	0.96
C8—C13	1.387 (2)	C17—H17C	0.96
C8—C9	1.391 (2)	C18—N1	1.311 (2)
C9—C10	1.390 (2)	C18—C19	1.503 (2)
C9—H9	0.93	C19—H19A	0.96
C10—C11	1.373 (3)	C19—H19B	0.96
C10—H10	0.93	C19—H19C	0.96
C11—C12	1.378 (3)		
			
N1—C1—C6	123.08 (13)	C11—C12—H12	120
N1—C1—C2	117.62 (14)	C13—C12—H12	120
C6—C1—C2	119.30 (14)	C8—C13—C12	120.70 (15)
C3—C2—C1	120.59 (16)	C8—C13—H13	119.7
C3—C2—H2	119.7	C12—C13—H13	119.7
C1—C2—H2	119.7	C7—C14—C18	120.33 (13)
C2—C3—C4	120.20 (16)	C7—C14—C15	120.08 (12)
C2—C3—H3	119.9	C18—C14—C15	119.58 (12)
C4—C3—H3	119.9	O1—C15—O2	124.63 (15)
C5—C4—C3	120.81 (15)	O1—C15—C14	124.46 (14)
C5—C4—H4	119.6	O2—C15—C14	110.91 (12)
C3—C4—H4	119.6	O2—C16—C17	107.69 (15)
C4—C5—C6	120.21 (16)	O2—C16—H16A	110.2
C4—C5—H5	119.9	C17—C16—H16A	110.2
C6—C5—H5	119.9	O2—C16—H16B	110.2
C1—C6—C5	118.89 (13)	C17—C16—H16B	110.2
C1—C6—C7	117.77 (12)	H16A—C16—H16B	108.5
C5—C6—C7	123.35 (13)	C16—C17—H17A	109.5
C14—C7—C6	117.96 (12)	C16—C17—H17B	109.5
C14—C7—C8	122.12 (12)	H17A—C17—H17B	109.5
C6—C7—C8	119.89 (12)	C16—C17—H17C	109.5
C13—C8—C9	118.59 (14)	H17A—C17—H17C	109.5
C13—C8—C7	120.19 (13)	H17B—C17—H17C	109.5
C9—C8—C7	121.21 (13)	N1—C18—C14	122.27 (13)
C10—C9—C8	120.56 (16)	N1—C18—C19	117.00 (14)
C10—C9—H9	119.7	C14—C18—C19	120.73 (13)
C8—C9—H9	119.7	C18—C19—H19A	109.5
C11—C10—C9	120.00 (16)	C18—C19—H19B	109.5
C11—C10—H10	120	H19A—C19—H19B	109.5
C9—C10—H10	120	C18—C19—H19C	109.5
C10—C11—C12	120.20 (16)	H19A—C19—H19C	109.5
C10—C11—H11	119.9	H19B—C19—H19C	109.5
C12—C11—H11	119.9	C18—N1—C1	118.58 (13)
C11—C12—C13	119.94 (16)	C15—O2—C16	119.11 (13)
			
N1—C1—C2—C3	178.06 (15)	C10—C11—C12—C13	−0.8 (3)
C6—C1—C2—C3	−1.1 (2)	C9—C8—C13—C12	1.3 (2)
C1—C2—C3—C4	0.8 (3)	C7—C8—C13—C12	−177.08 (15)
C2—C3—C4—C5	0.1 (3)	C11—C12—C13—C8	−0.5 (3)
C3—C4—C5—C6	−0.6 (3)	C6—C7—C14—C18	−0.5 (2)
N1—C1—C6—C5	−178.56 (13)	C8—C7—C14—C18	177.58 (12)
C2—C1—C6—C5	0.6 (2)	C6—C7—C14—C15	178.33 (11)
N1—C1—C6—C7	0.9 (2)	C8—C7—C14—C15	−3.6 (2)
C2—C1—C6—C7	−179.97 (12)	C7—C14—C15—O1	−76.8 (2)
C4—C5—C6—C1	0.3 (2)	C18—C14—C15—O1	102.0 (2)
C4—C5—C6—C7	−179.12 (14)	C7—C14—C15—O2	102.89 (16)
C1—C6—C7—C14	−0.56 (19)	C18—C14—C15—O2	−78.24 (17)
C5—C6—C7—C14	178.85 (13)	C7—C14—C18—N1	1.5 (2)
C1—C6—C7—C8	−178.70 (12)	C15—C14—C18—N1	−177.41 (13)
C5—C6—C7—C8	0.7 (2)	C7—C14—C18—C19	−177.80 (14)
C14—C7—C8—C13	−79.78 (18)	C15—C14—C18—C19	3.3 (2)
C6—C7—C8—C13	98.29 (17)	C14—C18—N1—C1	−1.1 (2)
C14—C7—C8—C9	101.92 (17)	C19—C18—N1—C1	178.13 (13)
C6—C7—C8—C9	−80.01 (18)	C6—C1—N1—C18	0.0 (2)
C13—C8—C9—C10	−0.8 (2)	C2—C1—N1—C18	−179.18 (13)
C7—C8—C9—C10	177.53 (15)	O1—C15—O2—C16	0.8 (3)
C8—C9—C10—C11	−0.4 (3)	C14—C15—O2—C16	−178.93 (15)
C9—C10—C11—C12	1.2 (3)	C17—C16—O2—C15	129.54 (19)

### Hydrogen-bond geometry (Å, °)

**Table:**

*D*—H···*A*	*D*—H	H···*A*	*D*···*A*	*D*—H···*A*
C16—H16A···O1	0.97	2.32	2.711 (3)	103
C19—H19B···O2^i^	0.96	2.52	3.374 (3)	147
C19—H19C···O2	0.96	2.59	3.212 (3)	122
C12—H12···Cg2^ii^	0.93	2.95	3.750 (3)	145
C17—H17C···Cg3^iii^	0.96	2.97	3.883 (3)	160

Symmetry codes: (i) −*x*+1, −*y*−2, −*z*+1; (ii) −*x*+2, −*y*, −*z*+1; (iii) −*x*+2, −*y*, −*z*.
